# Embracing the Emotion in Emotional Intelligence Measurement: Insights from Emotion Theory and Research

**DOI:** 10.3390/jintelligence11110210

**Published:** 2023-11-01

**Authors:** Marcello Mortillaro, Katja Schlegel

**Affiliations:** 1 Swiss Center for Affective Sciences, University of Geneva, 1202 Geneva, Switzerland; 2 Institute of Psychology, University of Bern, 3012 Bern, Switzerland

**Keywords:** emotion theory, emotional intelligence, ability EI, EI measurement, emotion regulation, emotion recognition ability, interpersonal emotion regulation, emotion understanding, emotion management

## Abstract

Emotional intelligence (EI) has gained significant popularity as a scientific construct over the past three decades, yet its conceptualization and measurement still face limitations. Applied EI research often overlooks its components, treating it as a global characteristic, and there are few widely used performance-based tests for assessing ability EI. The present paper proposes avenues for advancing ability EI measurement by connecting the main EI components to models and theories from the emotion science literature and related fields. For emotion understanding and emotion recognition, we discuss the implications of basic emotion theory, dimensional models, and appraisal models of emotion for creating stimuli, scenarios, and response options. For the regulation and management of one’s own and others’ emotions, we discuss how the process model of emotion regulation and its extensions to interpersonal processes can inform the creation of situational judgment items. In addition, we emphasize the importance of incorporating context, cross-cultural variability, and attentional and motivational factors into future models and measures of ability EI. We hope this article will foster exchange among scholars in the fields of ability EI, basic emotion science, social cognition, and emotion regulation, leading to an enhanced understanding of the individual differences in successful emotional functioning and communication.

## 1. Introduction

Over the past three decades, emotional intelligence (EI) has gained significant popularity as a scientific construct. It has entered the lexicon of everyday conversations to describe people who demonstrate adeptness or struggle when navigating emotionally charged encounters with others. Despite “rumors of the death” of EI in its early years due to problems with its conceptualization and measurement (Ashkanasy and Daus 2005), research in the field continues to thrive (e.g., Dasborough et al. 2022). However, the conceptualization and measurement of EI still face limitations, with many early criticisms (e.g., Locke 2005) remaining relevant today (Dasborough et al. 2022). For example, problems with defining objective scoring criteria and establishing construct validity in performance-based EI tests have already been discussed by Brody (2004), Geher and Renstrom (2004), Matthews et al. (2002), or Pérez et al. (2005).

In the present paper, we argue that this problem is still present and partly stems from a lack of theoretical foundation within existing EI tests. We propose avenues for future advancements in EI measurement by connecting some of the main EI components to models and theories from the broader emotion literature and by suggesting ways in which this literature can inform the development of novel and improved measures of EI.

Specifically, the present paper focuses on the assessment of ability EI, which is one of the two dominant EI approaches (see Fiori and Vesely-Maillefer 2018 for a review). Ability EI refers to a set of cognitive skills related to emotions, including “the ability to perceive emotions, to access and generate emotions so as to assist thought, to understand emotions and emotional knowledge, and to reflectively regulate emotions so as to promote emotional and intellectual growth” (Mayer and Salovey 1997, p. 5). Measuring such skills requires performance-based tests and emotion-related tasks with correct and incorrect (or more and less effective or adaptive) responses to capture “maximal performance.” For example, typical ability EI measures include judging which emotion was expressed in a picture or what action would best reduce one’s anxiety in a particular situation (situational judgment approach). 

In contrast, the second dominant EI approach refers to self-perceptions of emotional skills. Trait EI “essentially concerns people’s perceptions of their emotional world” and is rooted in personality research (Petrides et al. 2016, p. 335). Trait EI models vary substantially in the number and skills they consider and, therefore, each requires specific self-report instruments with items reflecting the skills included in the model. Nevertheless, all trait EI instruments target the test-takers’ propensity to behave in a certain way (“typical performance”, Sarrionandia and Mikolajczak 2020). This conceptualization requires self-report measures that present general context-free statements asking about people’s subjective self-perceptions. 

Though both trait and ability EI conceptualizations have advantages and limitations, researchers have highlighted that ability EI aligns more closely with the term EI (e.g., Cherniss 2010; Roberts et al. 2010). It maintains a narrower focus on emotions than the broader trait EI approach, which encompasses other concepts from positive psychology, including well-being and optimism. Additionally, ability EI is associated with intelligence, whereas trait EI is not (Roberts et al. 2010). Nevertheless, after three decades of research, only a limited number of scientifically validated ability EI tests exist. 

The most widely used test is the Mayer-Salovey-Caruso Emotional Intelligence Test (MSCEIT; Mayer et al. 2003), in which participants judge the appropriateness or effectiveness of actions or emotion labels in pictures or vignettes describing emotional situations. Other widely used tests are the Situational Test of Emotional Understanding (STEU) and the Situational Test of Emotion Management (STEM; MacCann and Roberts 2008). Like the MSCEIT, they use a situational judgment approach where participants choose an emotion label to describe an emotional situation (STEU) or an effective action for regulating an emotion in a vignette (STEM). Though several other ability EI tests exist, such as the Test of Emotional Intelligence (TIE; Śmieja et al. 2014), the Audiovisual Test of Emotional Intelligence (AVEI; Zysberg et al. 2011), and the Test of Emotional Intelligence (TEMINT; Blickle et al. 2011), they are notably less utilized (see review by Bru-Luna et al. 2021). More recently, the Geneva Emotional Competence Test for the Workplace (GECo; Schlegel and Mortillaro 2019) has been developed. 

The most common EI components across these tests are emotion perception/emotion recognition (the ability to identify and differentiate between emotions in oneself and others), emotion understanding (the ability to comprehend complex emotional states, transitions, and the causes and consequences of emotions), and emotion regulation/management (the ability to manage and respond to emotions in oneself and others effectively). These are the central ability EI components across different conceptualizations and taxonomies (e.g., Elfenbein and MacCann 2017; Mayer et al. 2016; Schlegel and Mortillaro 2019; Vesely Maillefer et al. 2018). 

As we will show in this article, a vast amount of the literature exists outside of the EI domain for each of these components, and the general emotion science literature can be readily linked to them. However, the ability EI conceptualization, research, and assessment developed independently from the general emotion science literature. Though this may sound surprising, we can suggest some reasons for this separation: different research methods (laboratory studies vs. testing), different goals (basic research vs. applied research), and a critical approach toward the concept of EI in the emotion literature. 

Nevertheless, this is probably one of the most surprising and unjustified separations between bodies of literature in psychology. Besides a few notable attempts (Fontaine 2016; MacCann and Roberts 2008; Peña-Sarrionandia et al. 2015), most empirical studies on EI and the measures they use refer only to other EI studies, with very little integration of the emotion literature despite that this problem was already pointed out more than twenty years ago (Matthews et al. 2002). With this paper, we would like to indicate how research on emotions and related fields can and should be the foundation of future EI assessments for each of these specific EI components.

## 2. Emotion Understanding

Emotion understanding competence refers to the ability to reason about the antecedents of the emotional experience and its implications for the person’s behavior. According to Mayer and colleagues (2016), emotion understanding is a higher-order competence that groups several areas of reasoning; among others, we can list labeling emotions and recognizing relationships among them, as well as appraising the eliciting situation, predicting how a person might feel in certain conditions, and recognizing cultural differences in the evaluation of emotions. 

### 2.1. How Definitions of Emotion Can Inform the Assessment of Emotion Understanding

Modeling emotion understanding and its measurement requires a clear and coherent theoretical framework that defines emotions, their components, and their implications. Unfortunately, this theoretical reasoning is often left implicit by researchers whose primary focus is creating a psychometrically sound measure. For example, in MSCEIT subtests for emotion understanding, the authors did not refer to any theoretical model to justify how they created the items and response options and how the correct response was defined. Concerning this last point, they relied on “expert scoring”, which is undoubtedly meaningful, but has several shortcomings, especially when experts are difficult to define or they disagree with each other (Barchard and Russell 2006). For these reasons, we think that theoretical grounding should be critical for building and scoring emotion understanding tests (see also Hellwig and Schulze 2021).

The emotion literature suggests three main theoretical views that can help define and measure emotion understanding. First, basic emotion theory (Ekman 1999; Keltner et al. 2019) is the approach used by most studies in emotion psychology and can be considered the standard in emotion recognition measurement, even in instruments that do not explicitly adopt this view. In a nutshell, according to this view, emotions are distinct categories, and it is possible to attribute a precise label to a specific emotional state. This conceptualization is implicit whenever one asks to label a scenario or an expression by choosing one particular emotion label. It is crucial, though, to understand that for many researchers, this is not an endorsement of the idea that emotions are universal and discrete, but a pragmatic way to access the knowledge about when emotions are experienced and how they are expressed. 

Second, dimensional theories of emotions propose that emotions can be understood and classified based on a small number of underlying dimensions. Russell (1980) introduced the circumplex model, which posits two primary dimensions in the emotional space: valence and arousal. Valence refers to the pleasantness or unpleasantness of an emotion, whereas arousal represents the level of activation or energy associated with it. Russell’s model suggests that a wide range of emotions can be mapped onto a circular space defined by these two dimensions. For instance, joy and love are located in the positive valence region, whereas fear and anger occupy the negative valence region. This model provides a foundation for understanding emotional experiences in a structured manner, but, to our knowledge, has never been used explicitly to assess emotion understanding. Still, it is not difficult to imagine researchers using this approach to build valid instruments. They could ask respondents to identify the valence and activation that one person may experience in the situation described in the item instead of asking to attribute an emotion label. A similar measure of emotion understanding may be simpler than the emotion labeling approach and valuable for clinical populations, young children, and in general in all those cases when labeling could be problematic (e.g., language difficulties, cultural variability).

Third, appraisal theory describes emotion as the result of a set of subjective cognitive evaluations that happen with or without awareness (Moors et al. 2013; Roseman 1996; Scherer 2001). In other words, it is not the events or the objects per se that elicit the emotion, but how one person appraises them. This subjectivity explains individual differences in emotional reactions, but also provides the basic framework to find commonalities between even very diverse experiences of one emotion. For example, anger can be characterized by an event appraised as goal-obstructive and unpleasant, likely caused by somebody (e.g., not casual, and due to chance), and for which the angry person has a high sense of coping. Given its flexibility and detail in explaining emotional experience, we think appraisal theory is the best candidate to model emotion understanding. 

### 2.2. Using Appraisal Theory to Asses Emotion Understanding

A few authors have used appraisal theory to create emotion understanding tests. MacCann and Roberts (2008) chose Roseman’s appraisal theory (Roseman 1996) for developing their Situational Test of Emotion Understanding (STEU). Roseman’s theory defines the appraisal profiles of seventeen emotions. Based on these theoretically predefined profiles, the authors created vignettes of emotional situations that became the items of the test. Answers are defined as correct or wrong depending on the theoretical pattern predicted by the theory. 

Similarly, the emotion understanding subtest of the Geneva Emotional Competence test is grounded in appraisal theory (Schlegel and Mortillaro 2019). In this case, the authors used the Component Process Model (CPM) of emotion (Grandjean and Scherer 2008; Scherer 2001, 2009). Like other appraisal models, the CPM identifies a set of appraisal dimensions that guide evaluating events and situations and generate specific emotional responses (Scherer 2001). These dimensions do not fully overlap with other models (e.g., Roseman’s), directly affecting how to develop the scenarios. In the GECo emotion understanding test, the items describe scenarios that reflect the collection of appraisals that characterize an emotion according to the CPM. For example, one scenario describes “John” attending an interesting presentation and being repeatedly disturbed by his neighbor who asks him questions. Regarding appraisals, the situation is moderately relevant, the other person’s behavior is obstructive but not intentionally harmful, and John has the potential to cope with the situation. This set of appraisals characterizes an experience of irritation. 

This way of measuring emotion understanding implies that emotion understanding involves perspective-taking and considering all the appraisals involved. Instead of directly attributing an emotional meaning to the event or situation, a person skilled in emotion understanding should be able to infer the likely appraisal process of the other person (Mortillaro et al. 2011). Is it something unexpected for them? Is it goal-conducive or goal-obstructive? Do they think that somebody else is responsible for it? Do they feel that they can cope with the situation? Being able to make these judgments accurately shows a high level of emotion understanding and would be a possibility for phrasing emotion understanding items. 

Emotion understanding in the sense of knowledge can also be measured for emotion components other than appraisals (Scherer 2009), including (1) physiological reactions that occur during emotional experiences; for instance, fear may be accompanied by increased heart rate and sweating; (2) expressive behavior, that is, the outward display of emotions through facial expressions, vocalizations, and body language; (3) action tendencies, that is, the behavioral inclinations or urges associated with specific emotions; for instance, fear may prompt a person to flee or avoid a threatening situation; (4) the subjective experience component, that is, the subjective and consciously “felt” aspect of emotions; for example, when feeling happy, an individual experiences a positive, pleasant subjective state. It is important to note that these components are interactive and interdependent, forming a dynamic system within the emotional experience. They influence and modulate each other, resulting in a coherent emotional response. 

Two recent measures demonstrated the feasibility of assessing knowledge about these four components in standard emotion understanding tests. First, the Geneva Emotion Knowledge Test (GEMOK; Schlegel and Scherer 2018) includes a subtest on measuring an accurate understanding of emotion blends through vignettes that systematically include information on all five emotion components (appraisal, expression, physiology, action tendencies, subjective feeling). It also includes a subtest that measures respondents’ accuracy in judging the likelihood of features (representing all five components) to occur when a specific emotion is experienced. Similarly, Fontaine and colleagues (Huyghe et al. 2022; Sekwena and Fontaine 2018) developed the Components of Emotional Understanding Test (CEUT), which consists of scenarios built based on the CPM and cross-cultural linguistic studies (Fontaine et al. 2007; Fontaine et al. 2013). For each scenario, participants rate the likelihood of several emotions, appraisals, action tendencies, bodily reactions, expressions, and subjective feelings. In the CEUT and GEMOK, participants must reason about the whole emotion process, making them excellent examples of how emotion theory can offer innovative ways to conceptualize and assess EI skills. This approach can be used to measure other under-assessed aspects of emotion understanding, such as knowledge about cultural differences (particularly in the expression component) and accuracy in predicting future emotions (affective forecasting) or emotion trajectories (Mayer et al. 2016). 

Recently, one more theoretical framework has been suggested for modeling and measuring emotion understanding: the empathic agent paradigm, consisting of two phases (Hellwig et al. 2020). In the first phase, test-takers learn about the emotion-related contingencies of a target person, that is, emotions, events, and actions. After this acquisition phase, the test takers apply this new knowledge to a novel situation involving the target person. This allows for objective scoring without assuming an absolute correct behavior, but only a more likely one based on contingencies. This approach tries to circumvent the problem of choosing a theoretical framework explicitly and, at the same time, not adopting a consensus-scoring approach. However, expecting an almost invariant behavior across similar situations for the same person implicitly assumes an appraisal approach (what matters is not the situation per se, but how the person appraises it).

## 3. Emotion Recognition

The ability to accurately recognize what another person is feeling from nonverbal cues (emotion recognition ability; ERA) is central to most ability-based theories, models, and taxonomies of EI (e.g., Mayer et al. 2016; Elfenbein and MacCann 2017; Vesely Maillefer et al. 2018). Specifically, ERA is assumed to contribute to the accurate understanding of the causes and implications of emotional situations (see previous section) and to the ability to influence what another person is feeling (see the section on emotion management). Perhaps because individual differences in ERA are assumed to be crucial for successfully navigating social interactions (for an overview, see Palese and Schmid Mast 2020), research on ERA and its assessment have had a long tradition dating back to the 1970s (e.g., Hall 1978). 

Despite the theoretical integration of ERA in EI models, the two constructs continue to be studied relatively independently. Research on ERA is scattered across different fields of psychology and comes with various and inconsistently used labels (e.g., emotion decoding, theory of mind, emotion perception, cognitive empathy). Other fields also tend to use different ERA measures (with their respective construct labels), and there have been only a few efforts to map the terrain of ERA assessment across domains. However, such integration is necessary for at least two reasons. First, ERA tests typically have low intercorrelations and, thus, do not measure one single skill (Schlegel et al. 2017). Second, most ERA tests have been constructed in a rather atheoretical fashion and reviewing them within the context of emotion and social perception theories can benefit the creation of new and improved assessment tools.

### 3.1. The Dominance of Basic Emotion Theory in ERA Measurement

Within the ability EI literature, the most common assessment of ERA is the MSCEIT Faces subtest^1^, in which participants rate the presence of several emotions in a series of photos of facial expressions. Most other standard ERA tests, however, use a forced choice format in which participants choose, out of a predefined set of emotion words, the option that best describes an emotional expression (typically, in a static picture of a face; for an overview, see Bänziger 2016). The expressions used in the MSCEIT and other tests are often posed by actors and limited to a few emotions. As a notable exception, the GERT consists of 14 emotions expressed by actors in videos with sound (Schlegel et al. 2014).

The widespread use of discrete emotion categories to create the stimuli and present the response options makes basic emotion theory (BET) the predominant theoretical framework for measuring individual differences in emotion recognition, that is, it is (implicitly) assumed that (facial) emotional expressions are readouts of discrete emotions with a fixed meaning and that emotions are decoded by matching sensory inputs of nonverbal cues with internal representations of distinct emotion categories, leading to the selection of the most likely emotion label (Dricu and Frühholz 2016). This approach also implies that individuals can have selective impairments in recognizing specific emotions—an idea widely studied in clinical research (e.g., Dalili et al. 2015). From a psychometric perspective, the BET approach to ERA testing has the advantage that the correct response for each item can be easily defined (it usually corresponds to the emotion the actor intended to portray). Additionally, a forced choice paradigm makes it easy to calculate ERA scores as the sum of correct choices and reduces testing time compared to rating scale items. However, the reliance on few emotion categories in terms of the stimuli and the dominance of the forced choice format have also sparked some criticism. 

### 3.2. Going beyond a Small Set of Basic Emotions and the Forced Choice Paradigm

Concerning the stimuli and emotions used, several scholars argued that in real life, people experience and express many more than just six or seven emotions and that naturalistic expressions rarely correspond to the prototypical portrayals used in standard stimulus sets (e.g., Matsumoto and Hwang 2017). In addition, using only a few response options makes some tests very easy, restricting the measurement of ERA in the higher ability range (Kenny 2013). 

Recent research on emotional expressions in the BET tradition provides a lot of potential for broadening the scope of ERA tests and increasing ecological validity. Several large-scale and cross-cultural studies have shown that perceivers can reliably distinguish 20 or more discrete emotions based on facial, vocal, and bodily expressions (e.g., Cordaro et al. 2020; Cowen et al. 2019; Cowen and Keltner, 2020). For example, Cowen and Keltner (2020) found that naturalistic facial–bodily expressions can reliably signal 28 distinct emotion categories. Although these studies do not focus on individual differences, their stimulus databases can likely be used to build new ERA assessments for different sensory modalities and a wide range of emotions. 

In a different attempt to go beyond prototypical emotional expressions, Israelashvili et al. (2021) created the Emotional Accuracy Test (EAT), which consists of four videos in which a young woman talks about an emotional life event. Test-takers rate each video on ten emotions, and ERA performance is calculated as the absolute difference between the participant’s and the target’s own ratings on each emotion. The EAT has demonstrated strong correlations with established ERA tests, showing that using naturalistic expressions with verbal content without defining a single correct answer is a viable approach to ERA measurement. 

Using emotion rating scales like in the EAT has also been suggested by others as an alternative to forced-choice testing (Fontaine et al. 2022; Hess and Kafetsios 2021). One obvious advantage is that it allows assessing accuracy in perceiving blends and complex affective states. In addition, Kafetsios and Hess (2023, in this special issue) have argued that even for “classic” pictures of discrete facial expressions, rating scales yield meaningful psychological information beyond the traditional “percent correct” score. Specifically, this format allows distinguishing “accuracy” (intensity of target emotion) from “bias” (intensity ratings on all non-target emotions) in line with the truth and bias model of person perception by West and Kenny (2011). According to this approach, participants can be both “accurate” in detecting a target emotion and have a “bias” towards perceiving emotions not present in the stimulus. An open question is whether this model and scoring format yields meaningful information when applied to more naturalistic expressions where no clear-cut target or “correct” emotion is present. 

### 3.3. Going beyond Emotion Words

Nevertheless, like more traditional ERA assessments, the EAT and other tests using emotion rating scales face another potential limitation of BET—the reliance on emotion categories. One problem with using emotion words is that their underlying meaning may differ between cultures, languages, or even age groups (Barrett et al. 2007; Hoemann et al. 2021). For example, in the GERT, English, French, and German speakers vary in their accuracy rates for sadness, despair, and anger, which might reflect cultural differences in the expression of emotions or differences in the meaning of the respective words (response options) in each language (Schlegel 2013). 

According to the circumplex model of emotion (Russell 1980) and appraisal models (e.g., Scherer 2001), it would, therefore, be more appropriate to measure ERA in terms of accurate evaluations of underlying emotional dimensions (valence, arousal) and appraisals (goal conduciveness, coping potential, novelty, etc.) of the event preceding an emotional expression. According to the CPM (Scherer 2001; see also Fontaine 2016), it would also be meaningful to include ratings of action tendencies or physiological variables associated with nonverbal expressions (Mortillaro et al. 2011). 

Many studies have examined the meaning dimensions underlying emotion words and nonverbal expressions (e.g., Fontaine et al. 2013; Laukka et al. 2005; Mortillaro et al. 2011; Shuman et al. 2017) and the results have been successfully implemented in the measurement of emotion understanding (CEUT and GEMOK; see previous section). Still, standard ERA assessments have not yet adopted dimensional or appraisal theories of emotion. One reason against adopting this strategy might be that emotion categories seem to have more explanatory value than appraisal dimensions for large sets of naturalistic expressions, contradicting dimensional emotion theories. For example, Cowen and colleagues (Cowen et al. 2019; Cowen and Keltner 2020) found that appraisal dimensions captured less variance in categorical judgments of facial, bodily, and vocal emotion expressions than emotion labels. 

However, appraisal dimensions and other emotion components might be more readily inferred and gain explanatory power when the emotional expressions presented are more complex and embedded in a social context. One future avenue worth exploring would be to ask participants to rate appraisal dimensions underlying the emotional experience using naturalistic videos with affective content. 

In addition, when naturalistic videos are used, participants could also be asked to make more complex inferences about the stimuli, for example, about what is happening in the situation or about the relationships among the individuals in the situation (Keltner et al. 2019). In fact, such assessments would be similar to tests that are already used in the clinical social cognition literature, e.g., the Movie for the Assessment of Social Cognition (MASC; Dziobek et al. 2006) or the Reading the Mind in Films Task (Golan et al. 2006). Another recent video-based test asking participants to make complex inferences about the characteristics, causes, and implications of affective situations was developed by Dael et al. (2022) in the interpersonal accuracy field (Workplace Interpersonal Perception Skill test, WIPS). 

However, even though ERA tests with more diverse and complex response scales, including appraisals and other dimensions, would arguably capture emotion perception more ecologically, the definition of what precisely they measure may become blurred (e.g., can ERA be distinguished from emotion understanding?).

### 3.4. (Re)Defining the Scope of ERA Tests?

Recent theoretical approaches vary widely in the role attributed to social context during emotion perception, which has important implications for measuring individual differences in ERA. For example, in their theory of constructed emotion (TCE), Gendron and Barrett (2018) propose that the stimulus-driven process of perceiving and categorizing nonverbal expressions (see Dricu and Frühholz 2016) is far less critical in real-life communication than the prediction of upcoming sensory input based on the shared situational environment of interaction partners. According to these authors, emotion perception should (only) be studied in settings where conceptual systems of emotion expressers and perceivers dynamically interact. 

The empathic accuracy paradigm developed decades ago by Ickes (e.g., Ickes 2001) fits within this theoretical approach. In this paradigm, two interaction partners freely label their felt emotions when viewing a recording of their interaction. Then, they label their partner’s emotions while viewing the recording a second time. The degree of correspondence between self and partner ratings is used to measure empathic accuracy. However, this procedure is very time-consuming and cannot be used as a standard test in which all participants are exposed to the same items. Thus, in this form, the TCE seems incompatible with measuring individual differences in standardized assessments. 

In a more moderate approach, Kafetsios and Hess (2023, this issue; also, Hess and Kafetsios 2021) have also criticized current ERA tasks for not containing social context because stimuli usually show only one individual without situational information. In their view, existing tests lack validity because they capture cognitive rather than social perception skills. Indeed, context is an influential variable shaping emotion perception and judgment (e.g., Hassin et al. 2013). In order to “infuse” social context into ERA measurement, Kafetsios and Hess (2023) developed the Assessment of Contextual Emotions (ACE), in which participants rate the presence of several emotions in a still picture of a target person who is surrounded by two other individuals also showing an emotional facial expression. In the future, this approach could be extended to cover more emotions (the ACE stimuli are based on four emotions) and multimodal stimuli to enhance ecological validity. 

In a contrasting view, Fiori and colleagues (e.g., Fiori and Vesely-Maillefer 2018) emphasized the need to develop more measures of context-free “fluid” emotion information processing skills, such as the ability to make fine-grained discriminations among emotions presented in blends (Gillioz et al. 2023). These authors have presented the first evidence that context-free basic nonverbal processing skills might have incremental validity in explaining real-life outcomes above more knowledge-based facets of EI and emotion perception (Fiori et al. 2022). 

The above discussion highlights how theories of emotion and social perception can inform how ERA is conceptualized and measured beyond the EI literature. For example, depending on the adopted framework, ERA may be conceived as a set of basic emotion-processing abilities or complex language-dependent and prediction-based communication skills. Future developments in assessing ERA should be explicitly embedded in these frameworks, which will help identify the facets of emotion perception for which standard tests are missing (e.g., tests including social context). In addition, researchers using current ERA tests should be aware that most of them are implicitly based on BET and acknowledge the implications when interpreting their findings.

## 4. (Intrapersonal) Emotion Regulation

A necessary clarification should be made about the terminology that we use here. In the original ability EI model, emotion management refers to both interpersonal and intrapersonal emotion management (Mayer and Salovey 1997). However, the literature outside EI uses the term emotion regulation rather than emotion management, which can lead to confusion. Furthermore, we think that intrapersonal and interpersonal emotion management should be considered as two independent components. We suggest using the term “emotion regulation” for the ability to regulate emotions in the self, and “emotion management” for the ability to regulate emotions in others (Schlegel and Mortillaro 2019). This distinction is already apparent in the literature, where research on emotion regulation predominantly refers to internal cognitive processes, such as reappraisal or suppression, as strategies for self-regulation (for example, McRae 2016; Ochsner and Gross 2008). In contrast, emotion management in others (or interpersonal emotion regulation) primarily involves behavioral strategies that necessitate anticipating others’ behaviors and engaging in interactive processes. Though it is common for emotion regulation and emotion management to be required simultaneously in real-life situations, it seems preferable to consider the two forms as separate abilities and measure them separately. Recent studies show that these two competencies have low correlations, empirically supporting the conceptual distinction (Schlegel and Mortillaro 2019; Simonet et al. 2021; Völker et al. 2023). 

### 4.1. The Process Model of Emotion Regulation

Emotion regulation is considered one of the most critical EI skills, and hundreds of empirical studies contribute meaningful evidence supporting its relevance for well-being, positive life outcomes, and even health (Gross 2013; McRae and Gross 2020). Therefore, one would expect this literature to be crucial for studies focused on multi-branch EI models. Unfortunately, research on emotion regulation remained largely separated from general EI research, as discussed in recent work by Peña-Sarrionandia and colleagues (Peña-Sarrionandia et al. 2015). These authors made a remarkable effort to reconcile these two bodies of literature and highlighted the need for theoretically grounded instruments.

The Process Model of Emotion Regulation is currently the most largely supported model of emotion regulation (Gross 2013; McRae and Gross 2020; Ochsner and Gross 2008). This model postulates that individuals employ various strategies to influence their emotions’ intensity, duration, and expression. It identifies different moments when regulation strategies can be applied: focused on the antecedent or the response. Antecedent-focused strategies involve modifying the initial emotional response, whereas response-focused strategies aim to regulate emotions after they have already been experienced. Five strategies are part of this model: (1) *Situation Selection*: at this initial step, individuals can regulate their emotions by selectively choosing or avoiding certain situations or environments. For example, if someone is aware that a situation consistently triggers negative emotions, they may proactively avoid it to prevent emotional distress. (2) *Situation Modification*: in this step, individuals modify the specific features of a situation to regulate their emotions. It may involve altering the environment, adjusting the timing of an event, or changing the nature of the interaction to create a more desirable emotional experience. For instance, someone might request a change in their work schedule to reduce stress or modify the physical environment to enhance positive emotions. (3) *Attentional Deployment*: during this step, individuals, by focusing on specific aspects of a situation, can influence their emotional responses. For example, consciously shifting attention toward positive aspects of a situation or away from negative images can reduce the intensity of an unpleasant state. (4) *Cognitive Change* is related to the appraisal process and implies the ability to modify the interpretation or evaluation of a situation. This step involves cognitive reappraisal, where individuals reinterpret the meaning of an event to alter their emotional responses. For instance, perceiving a challenging task as an opportunity for growth rather than a threat can lead to a more positive emotional experience. (5) *Response Modulation* focuses on strategies to regulate emotions after they have been experienced, for example, by suppressing the outward expression of emotions.

It is essential to mention that the effectiveness of each strategy can vary depending on the situational demands and individual characteristics, and this variability can be the basis for assessing individual differences in emotion regulation competence (Gross and John 2003; Webb et al. 2012). 

### 4.2. Current Measures of Emotion Regulation

Some self-report questionnaires originated from the process model of emotion regulation. This group includes the Emotion Regulation Questionnaire that investigated the last two strategies of the model—reappraisal, for cognitive change, and suppression, for response modulation (Gross and John 2003)—and the Cognitive Emotion Regulation Questionnaires that focuses on adaptive and maladaptive cognitive strategies used to regulate negative emotions (Garnefski et al. 2001; see below for a description of the strategies). Until recently, though, not even self-report questionnaires mapped all possible strategies suggested in the theoretical model discussed above. Recent examples are moving in this direction; this is the case of the Process Model of Emotion Regulation Questionnaire (PMERQ), which investigates ten strategies covering all steps of the process model (Olderbak et al. 2022).

If we turn to performance-based tests, we typically find emotion regulation only as part of multi-branch assessments. The relative absence of stand-alone emotion regulation performance tests can be partly related to the difficulty of assessing what is mainly an intrapersonal skill through tests that ask about overt behaviors. This difficulty is evident if we look at the few available examples. In the “emotion management task^2^” of the MSCEIT, test-takers read a story about a person experiencing an emotion and decide how effective different behaviors are for regulating the emotion toward reaching a specific goal, e.g., reducing anger or prolonging joy (Mayer et al. 2003). The stories described in the items are varied, and it is possible to relate the response options to specific stages of the process model of emotion regulation described above; however, this is only a post-hoc interpretation, and there is no systematic application of the model in creating the response options (see also a similar post-hoc analysis of regulation strategies in Allen et al. 2015). A similar approach is used in the Ability Emotional Intelligence Measure (AEIM), another multi-branch performance test that includes subscales targeting emotion regulation (Warwick et al. 2010). Although the AEIM has been withdrawn from use by the authors because of methodological problems involved in its validation, it used an original approach. Specifically, respondents read four scenarios and evaluate how effective three possible actions are to increase, decrease, or maintain a specific emotion. Though both the MSCEIT and the AEIM use consensus scoring to determine the effectiveness of each proposed action, the AEIM additionally measures confidence with the selected choices. AEIM confidence ratings were weakly positively correlated with performance, intelligence, and empathy, leading the authors to conclude that such ratings may capture a separate factor, that is, individuals with higher confidence scores may be better able to regulate their emotions during emotion-related decision making, and, hence, measuring such scores can complement consensus-derived knowledge-focused scores (Warwick et al. 2010). Confidence ratings in ability EI assessments may also provide a link with trait EI, as trait EI measures often encompass self-evaluations of one’s performance and self-efficacy in dealing with emotions (Joseph et al. 2015). All in all, confidence ratings can be a useful addition to ability assessments, especially when responses are scored in a binary (correct/incorrect) format, but further investigation is needed. 

### 4.3. A Proposal for Future Performance Measures of Emotion Regulation

In most current measures, the authors’ expertise and consensus or expert rating fully guided the item construction and scoring procedure. However, ignoring theories and evidence from emotion regulation research is a missed opportunity for ability EI; this reasoning motivated a different approach in the subtest of emotion regulation of the GECo (Schlegel and Mortillaro 2019). Here, the focus is explicitly on one specific stage of the process, cognitive change, the one most directly linked to the quality of the emotional experience. As discussed before, indeed, appraisals are the main determinants of emotions, and from the perspective of emotion regulation, reappraisal is one of the most effective and beneficial ways to regulate emotions (McRae and Gross 2020; Uusberg et al. 2019; Uusberg et al. 2023). 

In line with other performance measures, the GECo uses scenarios and asks respondents to choose the option they consider most appropriate to reduce negative emotions. In contrast to other questionnaires, the GECo asks participants to select the two cognitive strategies they would most likely use in the scenario presented in the item. Critically, the test asks about “thoughts” instead of “behaviors”. The response options were systematically created based on the cognitive emotion regulation strategies framework proposed by Garnefski and colleagues (Garnefski et al. 2001). This theory informed the creation of two adaptive and two maladaptive options, as defined in this model. The respondents choose two options, and their responses are correct if they pick the adaptive ones. Across items, the test includes five adaptive emotion regulation strategies (*acceptance*, acknowledging and accepting the situation and one’s emotions without judgment or suppression; *positive refocusing*, deliberately redirecting one’s attention toward positive or neutral aspects of the situation; *putting into perspective*, gaining a broader perspective on the situation; *refocus on planning*, developing a plan of action; *positive reappraisal*, actively reframing or reinterpreting a situation to find positive or beneficial aspects within it) and four maladaptive strategies (*self-blame*, attributing responsibility solely to oneself; *other-blame*, attributing responsibility solely to others; *rumination*, repetitive and passive dwelling on negative thoughts; *catastrophizing*, magnifying or exaggerating the negative aspects of a situation).

This approach allowed scoring the items based on theoretical assumptions without relying on consensus and experts (although these two criteria were used during the validation process). Similarly, in their Emotion Regulation Profile Revised questionnaire (ERP-R), Nelis and colleagues (2011)^3^ present 15 vignettes and ask respondents to choose one or several of eight strategies considered more or less adaptive to achieve the regulation goal. Adaptive strategies include the behavioral display of positive emotions, mindfully savoring the moment, capitalization, and positive mental time travel, and maladaptive strategies include the inhibition of emotion expression, fault finding, inattention, and external attribution/nostalgia. Interestingly, the regulation goals covered in this questionnaire are both reducing negative emotions and enhancing positive emotions. This choice is linked to the emerging literature on the positive role of strategies like “savoring” (see the section on emotion management below). Although the ERP-R strategies refer to different stages of the emotion regulation process, they do not systematically map them as the PMERQ does.

Based on the advantages and limitations of the measures discussed above, we suggest that a performance-based measure of emotion regulation should ideally fully cover the process model of emotion regulation. It should include items for the different stages and response options that reflect engagement and disengagement strategies. The PMERQ is a recent example of a more comprehensive and theory-grounded measure of self-reported emotion regulation, and performance measures should take the same direction. Furthermore, future tests should consider that the effectiveness of regulation strategies can vary depending on the context (Ladis et al. 2022).

## 5. Emotion Management or Interpersonal Emotion Regulation

As stated above, existing ability EI tests (except the GECo) typically do not distinguish between the ability to regulate one’s own and others’ emotions. In the MSCEIT, emotion management is measured through vignettes of situations in which a person is experiencing a positive or negative emotion. Test-takers are then asked to rate, for each of several possible reactions, how helpful it would be for the person. The reactions combine various thoughts and behaviors and cannot be mapped onto a specific theoretical framework. Only a few vignettes describe situations in which someone else is experiencing an emotion that can be managed. As such, the MSCEIT focuses primarily on knowledge about successful emotion regulation in the self. The STEM uses the same approach.

In contrast, the GECo contains a subtest in which test-takers explicitly identify the most appropriate action to manage someone else’s emotions (e.g., a colleague’s sadness when missing a promotion). These actions were created to represent the five strategies of conflict management theory (Thomas 1992), including avoidance, accommodation, collaboration, compromise, and competing. Importantly, based on the situational features of the scenario (available resources, time, expected future events, etc.), each of the five strategies was defined as the correct one in some of the scenarios, rather than always defining collaboration or compromise as the “best” strategy. This theoretical framework is particularly suitable for workplace settings that the GECo targets, but, obviously, many more strategies for influencing what another person is feeling can be imagined. It would be desirable for future assessments to capture the breadth of available emotion management styles to help generalize findings beyond the narrow set included in the GECo. The goal of this section is, thus, to review how theories and research outside the EI field can be harnessed to create new measures of the ability to manage others’ emotions. 

### 5.1. Extending the Process Model of Emotion Regulation to Interpersonal Emotion Regulation

As a straightforward extension of the previous chapter on intrapersonal emotion regulation, the model by Gross and John (2003) can also be adapted to the management of others’ emotions (Little et al. 2012). Specifically, the emotional experience of others, such as work colleagues or subordinates, can be influenced by interpersonal situation selection (e.g., creating an external environment to prevent stressful situations for others, e.g., by adjusting deadlines, delegating tasks differently), situation modification (e.g., alleviating the impact of stressors for others by offering assistance for meeting a deadline), attentional deployment (e.g., helping a disappointed colleague to focus their attention on a positive achievement), cognitive change (e.g., guiding a person to reframe negative thoughts or beliefs), and response modulation (e.g., comforting another person through appropriate nonverbal expressions). As for emotion regulation in oneself, emotion management strategies used in each of the five stages can be engagement- or disengagement-oriented, with engagement-oriented strategies expected to be more effective (Olderbak et al. 2022). 

Though Little et al. (2012) developed a self-report questionnaire of people’s tendencies to manage others’ emotions in the workplace at each stage (Interpersonal Emotion Management Scale, IEMS), this model could also be used for creating standard assessments to measure the ability to choose the most effective strategy in a given context. A promising way would be to create vignettes of emotional situations with specific situational characteristics that are theoretically well suited to each of the five regulation stages, similar to the approach taken for the emotion management subtest in the GECo (see above; Schlegel and Mortillaro 2019). This would accommodate the increasing evidence that many emotion regulation strategies are not uniformly “good” or “bad” across all situations (e.g., Brockman et al. 2017).

### 5.2. Co-Enhancing and Co-Dampening as Adaptive and Maladaptive Emotion Management Styles

Though the process model of emotion regulation is typically applied to negative emotions, a different line of research has coined the terms “enhancing” or “savoring” and “dampening” for regulatory responses to positive affect. *Enhancing* involves intentionally amplifying and prolonging one’s own positive emotions, whereas *dampening* downgrades or diminishes the positive experience, for example, by minimizing its importance (Feldman et al. 2008; Quoidbach et al. 2010). Generally, enhancing is positively associated with well-being, while dampening has been linked to lower well-being and depression (Quoidbach et al. 2010). Whereas most research has focused on these constructs in relation to one’s own emotions, Bastin et al. (2018) have examined them within the context of dyadic peer relationships. Specifically, they defined co-enhancing as jointly elaborating on and celebrating each other’s positive emotions within a relationship, fostering shared joy and deepening the emotional bond.

In contrast, they defined co-dampening as downgrading discussions of positive emotions in a dyadic relationship, potentially undermining the positive impact of shared experiences and relationship satisfaction for both individuals involved. Bastin et al. (2018) also developed the Co-Dampening and Co-Enhancing Questionnaire (CoDEQ), which asks about the frequency with which dyad members engage in specific behaviors associated with the two styles when one of them feels happy (e.g., “we talk about how proud the person who is happy can be”, “we remind each other that happy feelings don’t last”). Given that (co-)enhancing and (co-)dampening are conceptualized as adaptive and maladaptive, respectively, these styles and the specific behaviors in which they manifest (see also Quoidbach et al. 2010) could be used to measure emotion management ability specifically in response to positive situations. For example, similar to the GECo emotion regulation subtest, two behaviors reflecting each style could be used as response options in vignettes, and participants could be asked to choose the two options that best reflect what they would do. Each selected behavior corresponding to co-enhancing would be scored with one point. 

### 5.3. Other Strategies for Influencing People’s Emotions

Various other strategies for influencing what others are feeling have been examined in different fields of psychology, although these efforts remain to be integrated (e.g., Niven et al. 2009; Nozaki and Mikolajczak 2020). Recently, Xiao et al. (2022) have examined high- and low-engagement strategies for managing others’ emotions (labeled “extrinsic emotion regulation”). These include downward comparison, expressive suppression, humor, distraction, direct action, reappraisal, receptive listening, and valuing. Some of these strategies, although without the systematic distinction between high and low engagement, have also been included in a widely used self-report questionnaire measuring the regulation of one’s own and others’ emotions, labeled intrinsic and extrinsic emotion regulation (Emotion Regulation of Others and Self (EROS) scale; Niven et al. 2011). With a newly developed questionnaire, Xiao and colleagues (2022) showed that the MSCEIT positively correlated with three high-engagement processes (reappraisal, receptive listening, and valuing) and negatively correlated with two low-engagement processes (downward comparison and expressive suppression). These results suggest that high-ability EI individuals are willing to engage in effortful emotion management processes. As this research allows distinguishing between more and less adaptive management strategies (adaptive in the context of enhancing well-being and relationship quality; MacCann et al. 2023), it could also be used to create and score situational judgment response options in ability EI measures.

Going beyond the use of single emotion management strategies, some authors have also examined the perceived quality of different strategy sequences. For example, Feng (2009) found that emotion management efforts were perceived as more effective when they followed a sequential pattern of problem inquiry, problem analysis, emotional support, and advice giving than when they did not follow this order. Future EI tests could thus probe test-takers’ knowledge and use of such patterns. 

Though the emotion management/interpersonal emotion regulation literature typically focuses on strategies involving verbal behavior (e.g., humor) and complex actions (e.g., modifying a situation), a person’s emotions can also be influenced through nonverbal behaviors such as facial and vocal expressions and touch (e.g., Debrot et al. 2021). To date, individual differences in using such nonverbal behaviors have not yet been examined within the context of EI and emotion management. Therefore, a promising future avenue would be to develop predictions about more and less “adaptive” nonverbal behaviors within emotional encounters and incorporate them in video-based responses to situational vignettes. These responses, depicting people’s attempts at managing another person’s emotion, could differ only in their nonverbal, but not their verbal, content. Test-takers would then be asked to select the most effective response. 

### 5.4. Focusing on Different Preferences of the “Target”

Whereas the above literature assumes that some regulation strategies are generally more adaptive than others, other research highlights that the “target” individuals in the management process can differ in the strategies they prefer others to use. For example, Liu et al. (2021) examined the perceived helpfulness of 13 emotion management strategies in romantic partners which were classified as problem-oriented (e.g., reappraisal, problem-solving, and blaming) versus emotion-oriented (e.g., encouraging sharing, affection, emotion invalidation) and as supportive versus unsupportive. Their results showed that people differ in the strategies they prefer their partner to use in different situations. Similarly, Williams et al. (2018) showed that people differ in their tendency to seek social support in response to emotional events and in the extent to which they perceive social support as helpful. 

Therefore, future emotion management tests could consider incorporating information about the target person’s strategy preferences to measure test-takers’ sensitivity in identifying and flexibly applying different management strategies. Similarly, the behavioral adaptability model suggests that emotionally intelligent individuals should be able to adapt their behaviors to the different needs and traits of the interaction partner (Carrard and Schmid Mast 2015; Palese and Schmid Mast 2022). Supporting the need to include behavioral flexibility and adaptability when managing others’ emotions in standard EI assessments, this group of researchers found that individuals with higher ERA displayed higher behavioral adaptability to subordinates’ preferences when in the role of a leader (Schmid Mast and Hall 2018). 

## 6. Summary and Discussion

The aim of this article was to connect multiple fields and research lines within the broad domain of emotional functioning that rarely “talk” to each other and cite their respective works. As we discussed here, the creation of future ability EI assessments and the field of EI in general can benefit from the vast literature and recent developments in research on emotion, emotion regulation, and social cognition. The main recommendations and possibilities for ability EI test development addressed in this paper are summarized in Table 1. 

With respect to emotion understanding and emotion recognition ability (ERA), the review focused on the three prevailing paradigms in emotion science (basic emotion theory, dimensional/constructivist models, and appraisal models). Substantial progress has been made in assessing emotion understanding in recent years, with various authors proposing innovative approaches rooted in theory. As we look to the future, a promising next step would involve integrating the componential approach within a more contextualized framework that seeks to evaluate the process of understanding emotions, their evolution, and the intricate interplay between different emotional states. It is crucial to acknowledge the role of cross-cultural variability in future tests of emotion understanding, particularly when considering a constructivist or appraisal perspective. For example, behaviors deemed norm violations in one culture, likely triggering anger, may be acceptable in another cultural (or organizational) contexts and fail to elicit any emotion. Incorporating cross-cultural factors is also imperative for the development and validation of new ERA tests. Despite support for modern BET, there is also clear evidence for nonverbal dialect theory (Elfenbein 2013), indicating that emotion expressions are more challenging to decode when the target and perceiver come from different cultures. 

Turning to emotion regulation and emotion management, our review encompasses the process model of emotion regulation and its extensions to interpersonal regulation processes. Furthermore, we explored recent research aimed at identifying and measuring specific adaptive and maladaptive regulation strategies, such as engagement versus disengagement-focused strategies, and how these findings can inform the development of performance-based tasks. Advancements in the field highlight the need for new tasks that explicitly consider contextual factors, which can be easily manipulated within situational judgment items. In the case of emotion management, it is also crucial to account for differences in target characteristics, such as individual preferences, to achieve a comprehensive assessment.

Though the present article focused on each of the four components separately, the measurement of ability EI would, ultimately, also benefit from theoretical efforts to connect the single competencies. Although with the cascading model of EI (Joseph and Newman 2010), a starting point has been made to connect the ability EI branches, the most recent version of the ability EI model, as well as other ability EI conceptualizations (e.g., Elfenbein and MacCann 2017; Fiori et al. 2022), focus on a taxonomy of skills and do not specify the process through which they are potentially linked. 

A process model of EI should also examine the motivational and attentional aspects of emotionally intelligent behavior, which are likely to determine whether and how individuals use their maximal performance (which is what ability EI tests usually measure) in real-life settings. For example, some individuals with ERA scores may not pay much attention to others’ nonverbal behavior in everyday life and will, thus, not be able to fully use their ERA skill. Research on individual differences in “emotional attunement” is still in its infancy (Schlegel 2020). Further, there has been evidence for “motivated inaccuracy” in recognizing others’ emotions when accurate perception might harm a relationship (Simpson et al. 2003). Finally, a process model of EI should consider the mental effort required for emotionally intelligent behavior. For instance, Niven (2017) emphasized that managing others’ emotions may be depleting to perform and that some strategies tend to be particularly costly in terms of resources (cf. the distinction between high- and low-engagement strategies above; MacCann et al. 2023). 

Future research should, therefore, examine individual differences in the perceived levels of effort involved in each of the steps of the emotional communication process—paying attention to one’s own and others’ emotions, decoding emotional information, and engaging in different regulation and management strategies. Perceived effort, in combination with context-dependent motivational factors, may help explain the discrepancies between maximal and typical performance (Freudenthaler and Neubauer 2007). 

Future ability EI assessments should also consider culture’s role in shaping emotionally intelligent behavior when using tasks like the ones we described for emotion understanding. Assuming that we can confidently say that an intentional goal-obstructive behavior by somebody should likely elicit anger in individualistic cultures, this may differ in collectivistic cultures where the cost of social conflict may be higher.

Though situational judgment tests became a standard for ability EI measures, future measures should consider the specific social context in which they will be used. Several authors have argued that EI is not invariant across situations, for example, if we compare behavior in a family context to that in a work context (Jordan et al. 2002; Michinov and Michinov 2022). First, we can expect that the strategies employed for emotion regulation and management will differ depending on whether a person interacts with their supervisor or a six-year-old child. Second, we are likely better at handling the emotions of people we know better. One can more easily anticipate a close relative’s emotional reaction than a stranger’s in the same situation. Third, there is increasing evidence that most emotion regulation strategies are not inherently good or bad, but vary in their adaptiveness across situations, contexts, and people (Brockman et al. 2017). 

Last but not least, technological innovation could become an important asset for future assessments. To our knowledge, for example, no performance test can measure emotion expression and rate the extent of a successful “suppression” strategy or the ability to deliver a chosen emotion management strategy effectively (but see Olderbak et al. 2021). With the rise of AI technology, future assessments might also consider recording participants’ written or video-recorded reactions to emotional scenarios and automatically scoring these for emotion understanding or management (e.g., Schlegel et al. n.d.). 

### Towards a Chaos of Measures? A Glimpse into the Future of Ability EI Testing

Though existing ability EI tests will continue to be useful and have generated a large knowledge base about EI, many scholars emphasized the necessity for new measurement tools (for a discussion, see Dasborough et al. 2022). If our knowledge about ability EI is based on only a few tests, it will remain unclear whether the findings are due to the construct or the instruments (Roberts et al. 2010). 

We see at least two complementary strategies to develop new ability EI instruments in a systematic fashion. First, new tests might be developed for facets of EI branches that have been neglected by existing tests, such as the aptitude for expressing emotions or the understanding of cultural variations in emotion expressions and display rules. This approach would allow measuring the theoretical domain of EI more comprehensively, facilitating an exploration into which facets or branches are most predictive of central life outcomes or behavioral patterns. 

The second strategy might focus on creating batteries of tests for all EI branches rather than focusing on single branches and their subfacets. Though this second approach would likely aim for a unidimensional structure within each branch/subtest to facilitate the scoring and interpretation of the test scores, it would be advisable to base the item creation within each branch on more than one theory to cover each branch more broadly. For example, a new subtest to measure emotion management/interpersonal emotion regulation could cover the strategies from Gross’ (Gross and John 2003) model, as well as the high- and low-engagement strategies proposed by Xiao et al. (2022) and other strategies based on nonverbal behavior, as discussed above. 

The two approaches could collectively streamline research into the factorial structure of EI, as exemplified by Simonet et al. (2021) or MacCann et al. (2014). Drawing parallels from the history of cognitive ability testing, this process is likely to trigger several cycles of creating new test generations, evaluating their intercorrelations and structure, testing their validity, and refining or developing new tests. Although it will take time, we believe that this process is necessary to move the field forward. 

If we venture a glimpse into the future of ability EI testing, it is conceivable that increased efforts to build new tests (especially for under-assessed facets like expressivity) will result in the fractionation of EI. Although new tests like the GECo and GEMOK correlate highly with established tests (Schlegel and Mortillaro 2019; Schlegel and Scherer 2018), we know that measures within the emotion recognition branch show low intercorrelations (Schlegel et al. 2017), and for the intra- and interpersonal emotion regulation branches, the internal structure is still unknown, as there are only very few existing tests. Thus, we think that Elfenbein and MacCann’s (2017) description of ability EI as an umbrella term for a set of related, but distinct, skills may be fitting in the future when more tests are available. It is also likely that the different branches or subfacets differentially predict outcomes. For example, the literature already suggests that emotion management predicts wellbeing (MacCann et al. 2020), whereas this does not seem the case for emotion recognition (Schlegel 2020). 

But will a fractionation into more branches and subfacets with many tests and potentially different areas of predictive relevance be problematic for the field? We think that having a larger set of branches and/or subfacets under the broad ability EI umbrella need not result in chaos, provided there is a comprehensive theoretical framework to scaffold them, and assuming researchers reference the overarching construct, as well as the branch/facet labels they examine in their research to avoid ambiguity (for a similar discussion on the empathy construct, see Hall and Schwartz 2019). We also urge ability EI researchers to reference research from related domains as described above, and vice versa. Although the literature of individual EI domains like ERA or emotion regulation possesses distinct traditions and theories, we advocate that there is merit in unifying them under a broader EI label to better understand the entire process of emotional communication including its motivational and contextual aspects.

## Figures and Tables

**Table 1:** Avenues for the development of future ability EI assessments.

Emotion Understanding and Recognition	Relevant Citations
Incorporate knowledge about cultural differences (e.g., display rules)	(Mayer et al. 2016)
Assess understanding/recognition of emotion blends and transitions	(Schlegel and Scherer 2018)
Assess understanding/inferences about emotion components such as physiology or action tendencies and how they unfold in a target person	(Scherer 2009; Fontaine 2016)
Incorporate varying contexts and differences in target person’s characteristics; assess learning of new emotion-person contingencies rather than general knowledge	(Hellwig et al. 2020)
Use rating scales (e.g., for appraisal dimensions or for emotion labels) instead of forced choice format	(Fontaine et al. 2022)
**For emotion recognition specifically:**	
Use a wider range of emotion categories	(Cowen and Keltner 2020)
Use multimodal and/or naturalistic emotion expressions	(Schlegel et al. 2014; Israelashvili et al. 2021)
Incorporate social context into stimuli	(Hess and Kafetsios 2021)
**Emotion regulation and management**	
Apply strategies like the following to the regulation of own and others’ emotions; use them to create and score response options in situational judgment items:	
Situation selection/modification; attentional deployment; cognitive reappraisal; response modulation	(Gross and John 2003)
Engagement- and disengagement-oriented (or high- and low-engagement) strategies	(Olderbak et al. 2022; Xiao et al. 2022)
Acceptance, positive refocusing, putting into perspective, refocusing on planning, other-blame, self-blame, rumination, catastrophizing	(Garnefski et al. 2001)
Savoring, capitalization, mental time travel, inhibition, inattention, external attribution	(Nelis et al. 2011)
Enhancing and dampening of positive emotions	(Quoidbach et al. 2010)
Problem-oriented and emotion-oriented strategies	(Liu et al. 2021)
Assess regulation ability by emotion component (e.g., ability to regulate expressions, action tendencies, appraisals)	(Fontaine 2016)
Assess sequences of regulation strategies, e.g., combine regulation of own and management of others’ emotions in one scenario	
Assess and compare maximal performance (“which option is the best”) and typical performance (“what would you do”)	(Schlegel and Mortillaro 2019)
Vary effectiveness of regulation strategies by context and different needs/ preferences of the target	(Ladis et al. 2022)
Assess behavioral adaptability (flexibility in strategy selection and application)	(Palese and Schmid Mast 2022)

## Data Availability

Not applicable.

## Notes

1 The MSCEIT also includes a subtest to measure emotion perception in artwork—a skill which is rarely studied in the ERA literature and will, therefore, not be discussed here.

2 Despite the name «emotion management» (which here we use to refer to interpersonal emotion regulation), this task deals only with intrapersonal emotion regulation.

3 One may argue that expert scoring and theory-based scoring should give the same results. However, we think that at least two factors could lead to differing scoring keys: (1) most likely, experts make their judgment based on multiple theories and personal expertise/experience, making it difficult to know exactly why one answer should be considered correct; this is valuable, but it is not the same as theory-driven scoring for which there is a high degree of control; (2) theory makes one specific prediction, whereas within a sample of experts, one gets a varying degree of agreement and a binary (1/0) scoring key is less defensible.
